# The utility of muscle magnetic resonance imaging in idiopathic inflammatory myopathies: a scoping review

**DOI:** 10.3389/fimmu.2025.1455867

**Published:** 2025-01-27

**Authors:** Julie J. Paik, Lisa Christopher-Stine, Mikael Boesen, John A. Carrino, S. Peter Eggleton, Deborah Denis, Olga Kubassova

**Affiliations:** ^1^ Department of Myositis, Johns Hopkins University, Baltimore, MD, United States; ^2^ IAG, Image Analysis Group, London, United Kingdom; ^3^ Department of Radiology, Copenhagen University Hospital Bispebjerg and Frederiksberg, Copenhagen, Denmark; ^4^ Department of Radiology and Imaging, Weill Cornell Medicine, Hospital for Special Surgery, New York, NY, United States; ^5^ Global Clinical Development, Merck Serono Ltd., Feltham, United Kingdom, an affiliate of the healthcare business of Merck KGaA; ^6^ Global Clinical Development, EMD Serono Research & Development Institute, Inc., Billerica, MA, United States, an affiliate of the healthcare business of Merck KGaA

**Keywords:** dermatomyositis, idiopathic inflammatory myopathies, magnetic resonance imaging, myositis, polymyositis

## Abstract

Idiopathic inflammatory myopathies (IIMs) are muscle disorders characterized by proximal weakness of the skeletal muscles, inflammation in muscle, and autoimmunity. The classic subgroups in IIMs include dermatomyositis, inclusion body myositis, immune-mediated necrotizing myopathy, and polymyositis (PM). PM is increasingly recognized as a rare subtype and often included in overlap myositis, the antisynthetase syndrome when no rash is present, or misdiagnosed inclusion body myositis. Magnetic resonance imaging (MRI) has played an increasingly important role in IIM diagnosis and assessment. Although conventional MRI provides qualitative information that is helpful for diagnosis, its application for the quantitative assessment of disease activity is challenging. Therefore, advanced quantitative MRI techniques have been implemented in the past 10 years to highlight potential new applications of disease monitoring in IIM. The aim of this review is to examine the role of quantitative MRI techniques in evaluating the key imaging features of IIM, mainly muscle edema and muscle damage (fatty replacement and/or muscle atrophy).

## 1 Introduction

The diagnosis of idiopathic inflammatory myopathies (IIMs) has become increasingly clinico-serologic (1), and relies primarily on a combination of clinical features such as the pattern of weakness and objective findings such as muscle enzyme testing including creatine kinase, electromyography (EMG), muscle biopsy, and myositis autoantibody testing. Magnetic resonance imaging (MRI) is proven to be a valuable non-invasive tool in the assessment of disease activity and in helping to phenotype the various types of IIMs (2, 3). Using conventional MRI techniques (T2-weighted [T2w] imaging and short tau inversion recovery [STIR]), detection and evaluation of key features of pathological changes in patients with IIMs including muscle edema (ME), fatty replacement (FR) in muscles and muscle atrophy is based on visual assessment of gross morphologic and signal intensity changes (4, 5).

Conventional MRI techniques are limited to providing qualitative information. Advanced quantitative MRI techniques provide information that is important for the early detection of inflammatory changes and subtle changes in muscle injuries. These quantitative MRI techniques have shown promising results for evaluating ME, FR and muscle atrophy (6–8) by overcoming limitations in visual assessment, but most are not used routinely for the clinical evaluation of skeletal muscles.

MRI can also be used to identify a target site for muscle biopsy (9), while detection of specific MRI patterns of muscle involvement could help in IIM subtyping (3, 10). The level of inflammatory infiltrates within muscle biopsies has been shown to be significantly higher in patients with dermatomyositis (DM) and numerically higher in patients with polymyositis (PM) in areas of greater hypersignal intensity observed on fat-suppressed water-sensitive MRI images generated using STIR sequence (11). Using T1-weighted (T1w) and STIR MRI sequences of the lower limbs, Day et al. rated ME, FR and atrophy on a scale from 0 to 3 in a blinded retrospective review of MRI scans from 76 treated or potential IIM patients (10). Total edema and FR scores were significantly higher in patients with immune-mediated necrotizing myopathy (IMNM) compared with those with DM/PM and patients without IIM. Patients with inclusion body myositis (IBM) had high edema and damage scores, while those without IIM had low levels of edema, FR and atrophy. Review of serial MRI acquisitions showed subtype specific differences. Total atrophy and FR scores improved over time in most non-IBM patients while no improvement was seen in the patients with IBM (n=3). However, the authors indicated that inter-rater reliability for edema was fair to moderate, although higher for atrophy and FR (10).

In this article, we review the scope of currently available quantitative MRI techniques and their roles in the diagnostic evaluation, disease activity management and monitoring, and treatment response assessment of patients with IIMs. We focused on the evaluation of the main pathological features of IIMs: ME, FR and muscle atrophy.

## 2 MRI assessment in myositis

MRI provides high spatial and soft-tissue contrast resolution for the evaluation of individual muscles and muscle groups, and can be used to determine disease stage and severity (12, 13) in patients with genetic muscle diseases. It also allows both muscle morphological analysis (e.g., muscle atrophy) and muscle tissue characterization (e.g., FR and ME) (8). The proximal legs are examined most often because thigh muscles are commonly affected in IIM (4).

MRI assessment can be categorized into two main groups: semi-quantitative and quantitative methods. Semi-quantitative scoring systems are based on visual assessment by an evaluator, typically a radiologist, who assigns a numerical grade from a predefined scale, according to the disease extent and severity (6). These scoring systems have the advantage of being relatively easy to implement and use, and although they usually rely on subjective visual assessment of the muscle, studies have demonstrated good intra- and inter-observer agreement for each selected scoring system (14, 15) and are generalizable. Quantitative assessment methods use computer analysis of pixel intensity values, generating parametric maps that illustrate the various pathological processes occurring in skeletal muscle, including ME and FR (16, 17). These approaches provide sensitive and reproducible biomarkers, which are useful for follow-up studies (18, 19). To extract the relevant information within different regions of interest (ROIs), parametric maps must be combined with image segmentation. To extract quantitative information from parametric maps of disease muscle, ROIs should be manually or automatically defined on selected muscles (anatomical scan) so the diseased area can be precisely evaluated (20). In clinical studies, manual drawing of ROIs has been considered the gold standard (21, 22), but it is a time-consuming process, thus the use of automatic procedures would be advantageous to quantify the ME, FR and muscle atrophy in patients with IIMs (23–25).

### 2.1 Evaluation of muscle edema

**Figure 1:**
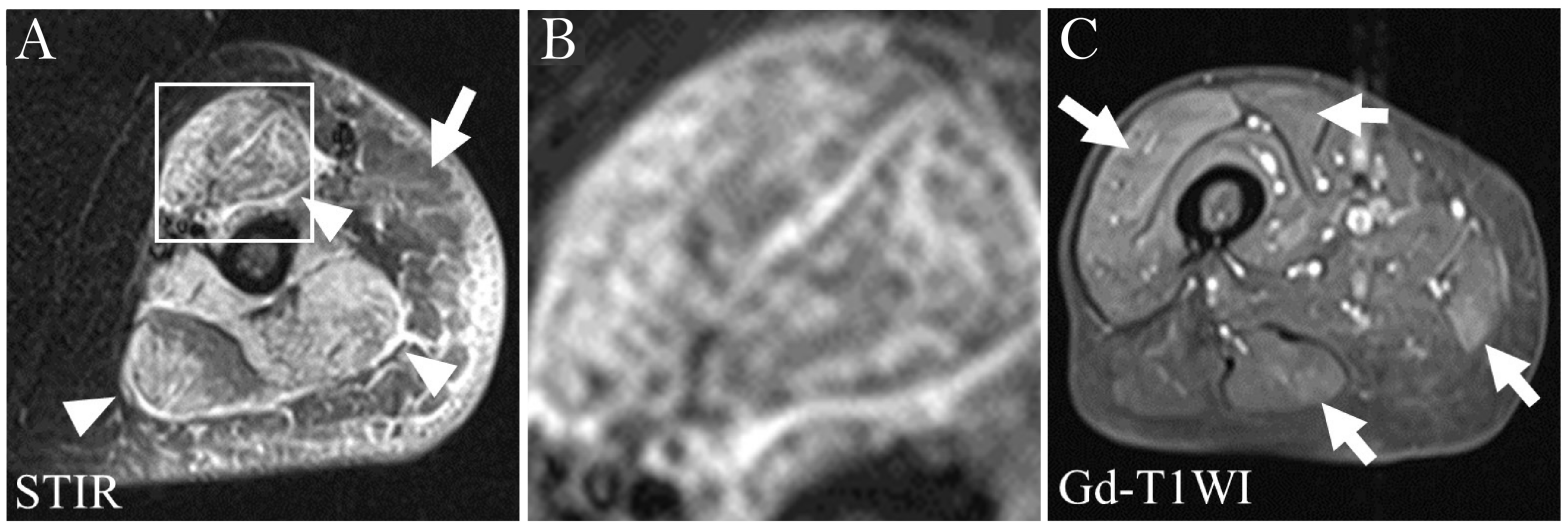
**(A)** STIR image of the left upper arm of a patient with DM. Structures with HSI are the subcutaneous adipose tissue (arrow), fasciae (arrowheads) and muscles. Distribution and pattern of HSI in the biceps muscle are diffuse and honeycomb, respectively (within the square). **(B)** Higher magnification image of the square in **(A)**. **(C)** Gd-T1WI image of the right thigh of a patient with PM. There is diffuse HSI in the vastus lateralis muscle and patchy HSI in the rectus femoris, gracilis and semitendinosus muscles, producing a foggy pattern (arrows). DM, dermatomyositis; Gd-T1WI, gadolinium-enhanced fat suppressed T1-weighted imaging; HSI, high signal intensity; PM, polymyositis; SHSI, strong high signal intensity. Figure adapted with permission from Ukichi et al. (31).

In pelvic and thigh musculature, bilateral, symmetric edema indicative of muscle inflammation is an early MRI finding in patients with PM or DM, typically resulting from an overall increase in water content (intracellular and/or extracellular) (26). Fat-saturated T2w or STIR sequences included in most thigh muscle scanning protocols can be used to evaluate ME. ME appears as a hyperintense signal that is more or less homogeneous without mass effect, whereas unaffected/normal muscles are typically homogeneous and appear as hypointense relative to both fat and water on non-fat-saturated T2w sequences (8, 27). STIR hyperintensity is typically interpreted as ME indicative of active inflammation in the muscle, leading to a potential diagnosis of IIM. However, in the absence of other diagnostic findings to confirm a diagnosis of IIM, diagnosis may be challenging as ME may be indicative of other disease processes in some clinical settings, including trauma, rhabdomyolysis, radiation therapy, and infectious myositis (26). Consequently, using MRI to guide muscle biopsy may increase diagnostic accuracy in determining whether observed ME is due to IIM (9, 11, 28, 29). STIR sequences are preferred because fat produces a bright signal similar to that of edema on T2w sequences whereas STIR sequences suppress the fat signal (low signal), leaving only edema as a source of hyperintensity (8). In addition, STIR allows for faster scanning and provides more homogeneous images. Fat-saturated T2w sequences have also been proposed to evaluate IIM disease activity. These sequences provide greater in-plane resolution than STIR and a faster scan time, particularly with newer artificial intelligence-driven reconstruction methods, but are more limited in larger field of view images, such as the pelvis, and are more sensitive to field inhomogeneities. While fat-saturated T2w sequences correlate very well with STIR sequences, they do not appear to provide a significant advantage over them (5). The use of these sequences also brings the possibility to assess the presence of fasciitis, which appears as a bright, thickened line bordering the muscles (30). The pattern of high signal intensity on STIR sequences was reported to associate with subgroups of IIMs (31). In patients with DM, high signal intensity is described as a heterogeneous reticular pattern within a muscle (‘honeycomb’ pattern), whereas in patients with PM it often has a ‘foggy’ pattern (**Figure 1**) (31).

The sensitivity of MRI in showing ME in myositis is high (80–90%), but the specificity of MRI is low, as ME can be encountered in other conditions such as myonecrosis, infection, neuropathy, denervation, rhabdomyolysis, ischemia, intense muscle exercise, and non-inflammatory myopathies (8, 32, 33). Many of these conditions may be associated with elevated serum creatinine kinase levels and raised inflammatory markers and may, therefore, mimic IIMs.

Semi-quantitative scoring systems have been developed in several studies to assess ME on fat-suppressed T2w or STIR sequences, but there is currently no standardized or fully validated technique (34). Stramare et al. suggested a six-point grading system for ME (0–5), considering the severity and localization of the inflammation (35). Many studies have demonstrated good intra- and inter-observer agreements for selected rating scales (36), nevertheless, visual assessment of intensity signal changes on fat-saturated T2w sequence is subjective and hard to quantify among different MRI scanners and acquisition parameters. Furthermore, Mankodi et al. demonstrated pathological changes (e.g. ME) can be measured using the tri-exponential model, which allows quantification of muscle water T2 independent of muscle fatty degeneration (37).

T2 relaxation time mapping is a quantitative MRI technique that measures T2 relaxation time in muscle tissue (38). This technique was shown to be an objective quantifiable method for determining pathologic FR of the muscles and for detecting very early microstructural changes in muscle; however, it should be noted that other disease conditions, ME for example, can also increase T2 relaxation times in muscle tissue (38). An earlier study (39) showed that while ME and FR occurred primarily in younger and older boys with Duchenne muscular dystrophy (DMD), respectively, both can coexist in a single patient. Consequently, an important consideration when applying the T2 mapping method to the data is to generate T2 maps with fat suppression so that the measured T2 relaxation times differentiate between increased T2 values due to ME and inflammation and those due to FR of the muscles (38). In addition to the quantitative aspects of T2 mapping, it can also be used to detect and confirm subtle abnormalities (40). A comparative study revealed significantly increased T2 values in patients with IIM (DM or PM) compared with healthy controls, and T2 values inversely correlated with muscle strength in both groups (15). T2 relaxation time may also be used to measure inflammation in muscle in patients with juvenile DM, which correlated with measures of muscle strength and function, and may have a role in the long-term follow-up of inflammation (41). For example, T2 values decreased after corticosteroid injection in some patients with DMD, although the results in this small group were variable, with some patients also showing increased or unchanged T2 values (42). Multi-exponential analysis of T2 relaxation time values showed that mono-exponential water T2 values were elevated in both patients with DMD and those with IBM but was unable to distinguish between them (43). However, T2 values proved to be more sensitive markers of disease presence and were able to distinguish between IBM and DMD. T2 relaxation time values also correlated with clinical weakness (43). Using the quantitative MRI technique, T2 maps, Ran et al. were able to show that T2 values in affected muscles of patients with DM were statistically significantly higher than those in the muscles of control patients without DM (44). Interestingly, it was also noted that unaffected muscles of patients with DM had higher T2 values than the unaffected controls, suggesting that MRI-derived T2 maps could detect potential subclinical abnormalities in muscles that appear unaffected on conventional MRI (25, 44).

Since inflammatory processes increase the water content in diseased muscles, diffusion-weighted imaging (DWI) can be helpful in the assessment of ME changes. DWI, a technique that can be used to assess muscles at microscopic scales, uses the Brownian motion of water molecules in tissue to derive a quantitative map, namely the apparent diffusion coefficient (ADC) (45). Areas of restricted water diffusion appear hyperintense (low ADC values), whereas areas of free water diffusion appear hypointense (high ADC values). DWI can be incorporated easily into standard MRI protocols, requiring only a few extra minutes of scan time (32). Quantification is performed at the volume element (voxel)-basis level; the intensity of each voxel has an assigned ADC value that allows for quantitative image analysis using ROIs manually drawn along the contours of the affected muscle (46). There is increased diffusion of water molecules within ME, which results in higher ADC values (47, 48). Several studies in patients with myositis have analyzed ADC values in muscle (46, 47, 49) and demonstrated higher mean ADC values in edematous muscles. Qi et al. studied edematous muscles of patients with DM and PM, and showed increased diffusive properties and increased capillary perfusion, as well as reduced perfusion volume compared with healthy individuals (47). In contrast, some edematous muscles, which have higher ADC values, did not show T2 hypersignals on STIR sequence, suggesting that DWI is more sensitive for detecting low-grade ME (49). ADC values have also been reported to correlate with myopathic findings on electromyography in myositis patients (46). Like conventional MRI, DWI techniques are not specific for myositis and abnormal results may be observed in other pathologies that are characterized by increased water content (49). DWI data may be useful in the longitudinal monitoring of disease activity in myositis, as one study reported normalization of DWI changes after treatment in a patient with DM (47). As such, this modality may have utility in the monitoring of disease activity, although the results should be replicated in a larger cohort.

Diffusion tensor imaging (DTI) can also be used to evaluate the anisotropic diffusion characteristics of tissues (48, 50). It has the advantage of measuring the directionality of skeletal muscle fibers (that are cylindrical and well-ordered along a specific direction), in which water molecules are most likely to diffuse. Based on this theory, DTI has also been applied in the evaluation of skeletal muscles with quantitative measurements of muscular microstructure and that are sensitive to structural differences between functionally different muscles (50, 51). DTI measurements were shown to have excellent test-retest variability and intra- and inter-rater variability for ROI mapping (52). A comparative study, performed by Farrow et al., showed no significant differences in the mean diffusivity and fractional anisotropy between patients with myositis and healthy controls in the hamstrings or quadriceps (15). A study in patients with DM, showed that both static and dynamic DTI could differentiate between patients with DM and controls, and that DTI scores correlated with T1 and T2 scores of disease severity (53). However, it should be noted that DTI is a relatively new modality used in the assessment of muscle diseases such as myositis that requires modern high-end MR scanners and special sequences and larger studies are needed to confirm these findings.

### 2.2 Evaluation of fatty replacement

ME is not the only pathological feature that is evaluated in IIM patients. FR may occur within the muscle due to chronic inflammatory processes. This pathological feature can be observed by MRI and is thought to represent irreversible damage, as opposed to disease activity. T1w turbo spin echo sequences without fat saturation are suitable for detecting the degree of FR/deposition in chronic inflammatory myopathies.

**Figure 2:**
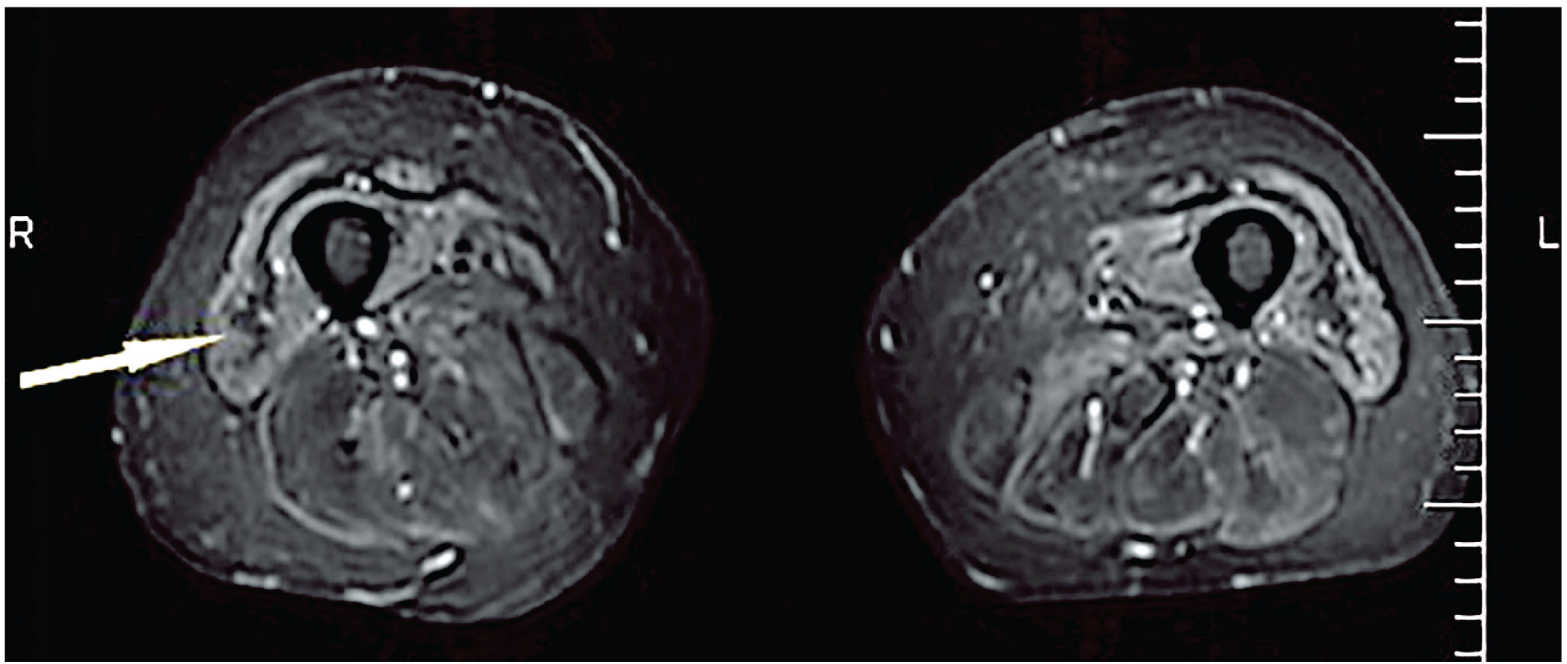
Undulating fascia sign (arrowed) observed in a patient with IBM on an axial STIR sequence. STIR, short tau inversion recovery sequence. Figure reproduced with permission from Kubínová et al. (58).

Semi-quantitative scoring of T1w images is used to assess the degree of FR in muscle; scores describe the severity of fatty involvement in individual muscles based on a visual assessment. The simpler T1w scoring of the degree of FR was used in a study by Cox et al., evaluating the utility of MRI in the diagnosis of sporadic IBM (54). In this study, muscle damage was graded as mildly, moderately, or severely abnormal, based on the extent of FR. Another scoring method, the Mercuri scale, can be used to evaluate FR in muscle by scoring T1w images (55, 56). This scale consists of six grades (Stage 0 to Stage 4, including Stage 2a and 2b) that range from normal appearance to a complete FR (55). FR followed by atrophy, which can be detected on T1w images as a typical “undulating fascia” sign, is mostly observed in IBM patients (57, 58) (**Figure 2**). Cox et al. showed that FR was the most common abnormality in patients with IBM (54), which contradicted the results from a previous study that reported ME and FR with almost equal frequency (59).

Other studies have used fat-corrected T2 maps of muscle where Dixon gradient echo techniques are used to generate fat maps that are subsequently subtracted from the T2 maps values to produce more valid muscle T2 values and quantify FR in muscle (60). These fat-corrected T2w values from multi-spin echo sequences have an advantage over T1w sequences for the quantification of fat, as they can be used to generate color-encoded T2 maps in which the pixel values represent the actual T2 transverse relaxation time (expressed in milliseconds). Segmentation of these T2 maps can be used to calculate average T2 values for different ROIs, which can subsequently be used to provide estimates of the fat fraction (FF) (38). In patients with muscle disease (IBM, facioscapulohumeral muscular dystrophy [FSHD], or oculopharyngeal muscular dystrophy), FR measured from quantitative MRI was found to correlate moderately with muscle histopathology, suggesting that MRI reflects structural changes in diseased muscle that could serve as an outcome measure for disease progression (61).

MRI-based FF measurements in the muscle are also useful for identifying FR or intramuscular fat (62, 63). One MRI technique that is being used more frequently to assess FR in myositis studies (27) is Dixon imaging (64, 65). This technique uses the phase difference between water and fat to separate these two components. Two different image weightings (two-point Dixon) can be acquired, to generate fat and water in- and out-of-phase images that can be used to quantify the amount of fat and water in the anatomy of interest. Recent technological advances have refined this technique with three-point and six-point Dixon that can further improve quantification and correct for non-uniformity in the magnetic field (66). From the image data, water images are generated by adding or subtracting the resultant in-phase or out-of-phase images. The intensity of each pixel in the resultant image is proportional to fat or water concentration. While the volume of fat and water within ROIs can be analyzed separately, it is a common practice to report the percentage of pixels classified as the FF (67).

### 2.3 Evaluation of muscle atrophy

Typically, T1w images are used to visualize muscle damage such as atrophy and FR (16, 68). In patients with IIM, muscle atrophy is usually associated with FR, but this atrophy can be symmetric or asymmetric and may also involve specific muscle subgroups. Atrophy is defined by a loss of muscular volume and is often determined by comparison with the contralateral muscle. However, it can be challenging to assess muscle atrophy by MRI at a single time point as the normative muscle size for a given muscle at a specific age and sex may not be available or reliable, especially if the disease has a symmetrical muscle involvement. Muscle atrophy may provide some useful information about disease progression if a patient is assessed longitudinally (10). In patients with IMNM, there was a trend towards more extensive ME (56%), atrophy (23%) and FR (38%) when compared with other subgroups of myositis (59). Almost all patients with IBM have muscle atrophy (54, 57), often at diagnosis (57), and that increases over time, but it is less diffuse than FR (54, 57). Furthermore, quantitative MRI techniques have shown that in patients with IBM there is progressive deterioration in muscle quality accompanying the loss of muscle mass (69). The degree of FR observed in patients with IBM has been correlated with declines in physical performance (69). The characteristic FR observed in patients with IBM may result from changes in ME resulting from inflammation (69, 70). Like ME and FR, muscle atrophy is not specific to IIM as it also occurs in chronic denervation and chronic steroid therapy (26).

## 3 MRI assessment in muscular dystrophies

Muscular dystrophies comprise a broad group of inherited conditions characterized by progressive weakening and atrophy of skeletal muscles, with MRI findings that are occasionally similar to those of IIMs, and therefore can be considered IIM mimics (71). Included in this group are the limb-girdle muscular dystrophies (LGMD) and Duchenne muscular dystrophy (DMD). DMD, the most common X-linked lethal childhood disease, results from a mutation in the gene encoding dystrophin, and is characterized by a progressive loss of muscle function and independent mobility (72, 73). An absence of functional dystrophin in the sarcolemma leads to reduced sarcolemma integrity, resulting in muscle fiber degeneration and progressive replacement of muscle by fat and connective tissue (74). The resulting changes in ME and FR can be evaluated using qualitative (T1 and T2) and quantitative (3-point Dixon sequences) MRI and magnetic resonance spectroscopy (MRS; e.g. single voxel ^1^H-MRS) techniques (75–78). These measures can be used to distinguish patients with DMD from healthy controls (76), and indicate the presence of inflammation, necrosis, damage and other disease processes that lead to increase in intracellular and/or extracellular edema in dystrophic muscles.

LGMDs are a heterogeneous group of disorders that comprise approximately 30% of all DMD cases (79), and includes dysferlinopathy (also known as LGMD type R2 or Miyoshi myopathy), an autosomal recessive disorder caused by mutations in the dysferlin gene, *DYSF*, which encodes a protein highly expressed in muscle and is essential in membrane repair (80). Dysferlinopathy is characterized by an active inflammatory and degenerative process leading to muscle fiber necrosis and replacement by fibrous and fatty tissue (81). This process is reflected in longitudinal MRI studies (82–84) showing that skeletal muscle water T2, which is sensitive to inflammation and edema and reflects disease activity, was significantly elevated in patients with dysferlinopathy (84), while there was a strong relationship between muscle FR (around 4% per year), disease duration, and function (84). Dysferlinopathy can be misdiagnosed as IIM, particularly IMNM, because patients have high creatinine kinase and proximal muscle weakness. In these cases, the degree of FR seen on MRI, which would be significantly greater in muscular dystrophies especially in the early stage of disease, can be helpful to distinguish from IIM.

Fukuyama-type congenital muscular dystrophy (FCMD) is an autosomal recessive disease, exhibiting muscular dystrophy, as well as central nervous system and ocular malformations (85) and is one of the most common LGMDs in Europe (86). FCMD results from mutations in the fukutin-related protein gene, a gene involved in glycosylation of α-dystroglycan, one of the components of the dystrophin-glycoprotein complex linking extracellular and intracellular proteins. MRI of the lower extremities of patients with FCMD using T1w sequences (87) or T1w sequences and 3-point Dixon sequences (88) demonstrated a distinctive pattern of muscle involvement with moderate or severe fatty infiltration in the adductor muscles, the posterior thigh and posterior calf muscles.

## 4 Discussion

Muscle MRI is an increasingly important tool for the diagnosis and assessment of disease activity and chronicity in IIMs. It is used to detect ME and FR that may aid the diagnosis and phenotyping of IIMs (13) (see **Table 1**). Conventional MRI techniques, such as axial T1w and T2w sequences with or without fat suppression as well as STIR, are the most frequently used imaging sequences to perform both morphological and tissue characterization of muscle. However, the variability in interpreting and scoring individual biopsy muscle abnormalities was found to be very high among experienced evaluators, suggesting the need for standardized and harmonized assessments for subgroup classification of IIMs (89).

**Table 1:** Overview of MRI techniques used in IIMs.

MRI technique	MRI findings	Strengths	Limitations
Muscle edema			
T2-weighted with STIR or SPAIR for fat suppression	T2w hyperintense signal without mass effect Primarily homogeneous	• STIR or SPAIR suppresses lipid signal, which also appears as hyperintense signals • Greater in-plane resolution versus STIR • Correlate well with STIR images • Faster scan time, particularly with AI-driven reconstruction methods • Semi-quantitative scoring systems developed to assess ME • Tri-exponential model can be used to quantify muscle water T2 independent of fatty degeneration	• Fat suppression required. On non-fat suppressed images, lipid hyperintensity is confounding • Quality control of fat suppression must be performed on subcutaneous tissue and bone marrow • ME may be indicative of other disease processes in some clinical settings • More sensitive than STIR to field inhomogeneities • Signal not necessarily homogeneous • Semi-quantitative scoring systems not fully validated
STIR sequences	STIR hyperintensity interpreted as active inflammation	• STIR sequences suppress fat signals – ME is the only source of hyperintensity • Provides more homogeneous images versus T2w • Can assess presence of fasciitis • Patterns of high signal intensity can correlate with IIM subgroups	
T2 relaxation time mapping	Increased T2 relaxation times in muscle reflect increased edema	• Objective, quantifiable method for determining FR and ME • Can detect and confirm subtle abnormalities not seen on conventional MRI • T2 relaxation time values have been correlated with muscle strength in patients with IIM (DM or PM)	• T2 relaxation time measures both ME and FR, which can occur in the same patient • Fat-suppression is required to differentiate between ME and FR • Other conditions may increase T2 relaxation times
Diffusion-weighted imaging	Areas of restricted water diffusion appear hyperintense Areas of free water diffusion appear hypointense	• Higher diffusion of water correlates with ME • DWI can be easily incorporated into MRI protocols • Requires only a few extra minutes of scan time • Quantitative analysis is possible • Longitudinal monitoring may be possible	• Technique not specific for myositis • Other diseases characterized by increased water content may give similar results
Diffusion tensor imaging	DTI images are used to determine ADC, eigenvalues and FA	• Measures the directionality of skeletal muscle fibers in which water molecules are most likely to diffuse • Static and dynamic DTI could differentiate between patients with DM and controls • DTI scores correlate with T1 and T2 scores of disease severity	• DTI is a relatively new modality used in the assessment of muscle diseases such as myositis • Requires modern high-end MR scanners • Special sequences and larger studies are needed to confirm these findings
Fat replacement			
T1w turbo spin echo sequences without fat saturation	FR appears as hyperintense signals Atrophy following FR shown as “undulating fascia” sign	• Detects the degree of FR/deposition in chronic inflammatory myopathies • Semi-quantitative approaches to determine severity can be used	• Severity based on visual assessment
T2w with Dixon gradient echo techniques (fat correction)	Dixon gradient echo techniques are used to generate fat maps that are subsequently subtracted from the T2 map values to produce more valid muscle T2 values and quantify FR in muscle	• Can be used to generate color-encoded T2 maps in which the pixel values represent the actual T2 transverse relaxation time and calculate average T2 values for different ROIs and estimates of FF	
Dixon imaging	Two different image weightings (two-point Dixon) can be acquired, to generate fat and water in- and out-of-phase images that can be used to quantify the amount of fat and water in the anatomy of interest	• Addition/subtraction of in-phase or out-of-phase images provides pixel intensities that are proportional to fat or water content • Newer three-point and six-point Dixon that can further improve quantification and correct for non-uniformity in the magnetic field	• Sequences are generally longer • Fat only images cannot provide the anatomical information contained in T1w images • Technique less reliable in inhomogeneous areas • Reconstruction artifacts, called “swapping artifacts”: Calculations converge to the wrong substance, producing a fat-only image when a water-only image is desired
Muscle atrophy			
T1w MRI	T1 hyperintensity in muscles indicates FR and atrophy	• Muscle atrophy may provide some useful information about disease progression if a patient is assessed longitudinally	• Muscle atrophy not specific to IIM • In patients with IIM muscle atrophy is usually associated with FR, but this atrophy can be symmetric or asymmetric and may also involve specific muscle subgroups • Assessment of muscle atrophy by MRI at a single time point is challenging as the normative muscle size for a given muscle at a specific age and sex may not be available or reliable, especially if the disease has a symmetrical muscle involvement

ADC, apparent diffusion coefficient; DTI, diffusion tensor imaging; FA, fractional anisotropy; FF, fat fraction; FR, fatty replacement; IIM, idiopathic inflammatory myopathy; ME, muscle edema; MRI, magnetic resonance imaging; ROI, region of interest; SPAIR, spectral attenuated inversion recovery; STIR, short tau inversion recovery; T2w, T2 weighted.

FR is reflected as increased signal intensity within the muscles on T1w images, whereas fat-saturated T2w and/or STIR images can help to visualize edematous and inflammatory changes in muscle, fascia and sub-cutis. The degree of FR in a specific muscle correlates with functional outcome measures in several muscle disorders such as DMD and FSHD, which has strengthened the use of quantitative muscle MRI as an outcome measure in clinical trials (90, 91).

Although there are patterns of muscle involvement between IIM subgroups and muscle MRI, there is a lack of longitudinal and comparative studies. Considering that atrophy and FR are features of the normal aging process, distinguishing these from IIMs, particularly in older patients, may pose a considerable challenge. Until recently most muscle MRI studies had a strong diagnostic emphasis focusing on correlations between IIM subgroups and characteristics on the radiological level. However, quantitative MRI techniques are now being used to evaluate key features of pathological changes (including ME, FR and atrophy) in patients with IIMs more precisely and consistently (8, 11, 59) and highlights the potential for muscle MRI to be utilized as a surrogate marker for monitoring disease progression and assessing therapeutic effects (92).

Muscle FF assessments (3-point Dixon technique) for quantifying FR in muscle in an objective manner (65, 75), and T2 relaxation time values (T2 maps) and Dixon techniques of separated fat and water components are currently being used as quantitative muscle imaging techniques, allowing comparisons between different patients and longitudinal assessment of the same patient over time. Accuracy of FF from the Dixon technique might be further improved by correcting for T2* and field inhomogeneities, accounting for noise bias, and using both three-point and more recently six- point Dixon multipeak models specific to skeletal muscle (93).

To improve diagnostic accuracy and speed up processing times, machine learning (ML) models are being applied to imaging data to aid in segmentation and the identification of ROIs in patients with IIMs. Nagawa et al. used an ML model to perform texture analysis (image quantification using pixel distribution and their surface intensity or patterns) on the muscles of patients with PM, DM or amyopathic dermatomyositis (ADM) and a control group of patients without IIMs (94). The authors demonstrated that ML-based texture analysis was able to distinguish between the different IIMs but was less accurate at distinguishing between the IIM and non-IIM disease groups (94). Similarly, Wang et al. were able to use a deep learning approach for segmentation of T2 maps in patients with IIM (DM and PM) to distinguish between patients with IIMs and those without (25). These data show that ML-based protocols have the potential to improve both the speed and accuracy of diagnosis, although further validation in larger studies will be required.

Another quantitative MRI technique currently being used for muscle assessment is MRS, which is suitable to measure water and fat concentrations (1H) and energy metabolites (31P, glycogen 13C) that may be relevant for certain metabolic disorders (95) but also some of the muscular dystrophies (96, 97). In quantitative MRI methods, ROIs drawn along the contours of the affected muscle can include both FR and ME, hence, these two pathologies may have an opposite influence in DTI measures, and reduce the sensitivity of disease measurement (47). DWI changes seen in one patient with IIM appeared to normalize with therapy in a longitudinal study (47). As such, this modality may have utility in the monitoring of disease activity, although these results need to be replicated in a larger study cohort. The use of fully quantitative sequences eliminates the potential bias introduced by subjective scoring systems. Furthermore, to overcome the inherent subjectivity of traditional scoring systems and conventional morphological parameters, radiomics imaging analyses (extraction of quantitative data based on the grey-level intensity) is being used in various diseases (98). This technique uses texture analysis to calculate entropy (heterogeneity) or uniformity within an image and may improve tissue characterization by detecting muscle features that cannot be perceived on visual inspection (99).

## 5 Conclusion

In summary, quantitative MRI is a promising technique that captures IIM disease severity. The use of fully quantitative sequences to evaluate ME, FR and muscle atrophy significantly reduces the potential biases introduced by more subjective scoring systems. Future longitudinal studies to assess treatment responsive in larger cohorts or clinical trials are needed to highlight quantitative MRI as a novel, powerful imaging technique in IIM.
